# Determination of ETV6‐RUNX1 genomic breakpoint by next‐generation sequencing

**DOI:** 10.1002/cam4.579

**Published:** 2015-12-29

**Authors:** Yanliang Jin, Xingwei Wang, Shaoyan Hu, Jingyan Tang, Benshang Li, Yihuan Chai

**Affiliations:** ^1^ Department of Hematology and OncologySoochow University Affiliated to Children's HospitalJiangsu215003China; ^2^ Department of Hematology and OncologyShanghai Children's Medical CenterKey laboratory of Pediatric Hematology and Oncology Ministry of HealthShanghai Jiao Tong University School of MedicineShanghai200127China

**Keywords:** Acute lymphoblastic leukemia, breakpoint, childhood, ETV6‐RUNX1, next‐generation sequencing

## Abstract

The t(12;21)(p13;q22) ETV6‐RUNX1 gene fusion is one of the most common chromosomal translocation in childhood acute lymphoblastic leukemia (ALL). It is associated with favorable prognosis. The identification of the genomic sequence of the breakpoint flanking regions of the ETV6‐RUNX1 translocation should be the best strategy to monitor minimal residual disease (MRD) in patients with ETV6‐RUNX1‐positive ALL. In this study, the ETV6‐RUNX1 translocation was sequenced by next‐generation sequencing (NGS) in 26 patients with ETV6‐RUNX1‐positive ALL and re‐sequenced by using the Sanger method. Interestingly, the three‐way translocation, including ETV6‐RUNX1, was detected in five patients. Four of them relapsed during or after therapy, while 21 patients without the three‐way translocation were still in remission (*P* < 0.0001). The three‐way translocation pattern was identical between the diagnosis and relapse samples in three patients, excluding one patient (SCMC‐001245). The relapse samples retained the translocation of ETV6‐RUNX1 relative to the three‐way translocation t(8;12;21) at diagnosis, suggesting that the three‐way translocation might be an important risk factor for relapse in patients with ETV6‐RUNX1‐positive ALL and should be further studied.

## Introduction

Childhood acute lymphoblastic leukemia (ALL) is a heterogeneous disease comprising different immunophenotypes and various genetic subtypes, including chromosomal translocations, single nucleotide variants, insertion/deletion (indel) of driver genes, and inappropriate expression of oncogenes 1, 2, 3, 4, 5, 6, 7, 8, 9, 10, 11. Primary genetic abnormalities can be detected in more than 75% of childhood ALL cases by standard genetic analyses. However, to date, the full repertoire of genetic alterations remains unknown 12. For B‐lineage ALL, hyperdiploidy (>50 chromosomes per leukemic cell) and the chromosomal translocation of t(12;21)(p13;q22), leading to an ETV6‐RUNX1 gene fusion, are the two major genetic subtypes. ETV6‐RUNX1 rearrangement results in the in‐frame fusion of the 5′‐region DNA‐binding domain of ETV6 to almost the entire RUNX1 locus. The expression of the ETV6‐RUNX1 chimeric transcription factor is considered to convert RUNX1 from a transcriptional modulator to a transcriptional repressor of RUNX1 target genes 13. The continuous expression of the ETV6‐RUNX1 influences the biology of leukemia subtypes and constitutes an essential driving force for the propagation and maintenance of the leukemic process 14. As a frequent and initiating event in childhood ALL, ETV6‐RUNX1‐positive preleukemic cells may originate in utero and suffer one or more subsequent genetic “hits,” inducing leukemia 15, 16, 17, 18, 19. The pathogenesis of ETV6‐RUNX1‐positive leukemia is complicated. It includes the loss of untranslocated ETV6 allele, the activation of the signal transducer and activator of transcription 3 (STAT3) by increasing RAC1 activity, the reduction of sensitivity to TGF‐beta‐mediated inhibition of proliferation, and the antiapoptotic action induced by the suppression of miRNA‐494 and miRNA‐320a 18, 20, 21, 22.

ETV6‐RUNX1 fusion protein is expressed in 25% of childhood B‐lineage ALL cases and is associated with favorable prognosis following conventional therapeutic strategies 23, 24, 25, 26, 27, 28. However, some patients relapse at a late stage during therapy or after therapy, while they could also achieve sustained second remission. Clone analysis by immunoglobulin heavy chain/T‐cell receptor (IGH/TCR) gene rearrangements, copy number variation profiling, and whole‐genome sequencing showed that, in ALL relapse, cells derived from the major or minor clones identified at diagnosis, which accumulated new mutations probably due to DNA damage caused by cytotoxic chemotherapy 18, 29, 30, 31, 32. The minimal residual disease (MRD) of these clones can be precisely determined by IGH/TCR rearrangement, flow cytometry, and chromosomal translocation, which can be used to precisely assess the early treatment response and predict early recurrence 33, 34, 35, 36. For ETV6‐RUNX1‐positive leukemia, the translocation of chromosomes 12 and 21 is one of the best targets to monitor MRD and is an accurate measurement of ETV6‐RUNX1 transcripts by quantitative real‐time polymerase chain reaction (Q‐PCR) 37, 38, 39, 40. However, ETV6‐RUNX1 transcriptional level in leukemic cells is unstable. Thus, it is difficult to detect the absolute number of leukemic cells by using methods such as flow cytometry, which allows the identification of one leukemic cell among 10,000 nucleated bone marrow cells or less 36, 41. The genomic flanking sequences of the breakpoint fusion for ETV6‐RUNX1 originate from chromosomes 12 and 21, which are not only the natural sequences of each leukemic sample, but also are the patient‐specific TEL‐AML1 genomic fusion sequence. Thus, they can be used to detect MRD by QRT‐PCR or other methods.

The ETV6‐RUNX1 translocation results from the break and rearrangement of chromosomes 12 and 21, two common translocation breakpoints have been described. The major breaking pattern involves a break within ETV6 intron 5 and RUNX1 intron 1, generating ETV6 intron 5‐RUNX1 intron 1 rearrangement. The minor breaking patterns are found in about 10% of ETV6‐RUNX1‐positive ALL and breaks within ETV6 intron 5 and RUNX1 intron 2 or 3, generating ETV6 intron 5‐RUNX1 intron 2/3 rearrangement 42, 43, 44. Theoretically, breakpoints are scattered within the 14.7 kilo base pairs (kbp) of ETV6 intron 5 and within more than 166 kbp of RUNX1 introns 1 to 3. Even though the distribution of ETV6‐RUNX1 breakpoints has the characteristic of significant microclustering in introns of ETV6 and RUNX1 in many patients, it is still difficult to identify or to sequence the breakpoints by streamlined long‐distance PCR or long‐distance inverse PCR 45, 46. In this study, we established a method based on next‐generation sequencing (NGS) to capture and sequence the breakpoint involved in ETV6‐RUNX1 translocation. Our method allows a rapid and accurate identification of the breakpoint.

## Materials and Methods

### Clinical sample collection

Bone marrow samples from newly diagnosed patients with childhood ALL were collected from the Department of Hematology and Oncology, Shanghai Children's Medical Center (SCMC). All samples were ETV6‐RUNX1 positive as determined by reverse transcription PCR or by fluorescence in situ hybridization (FISH) from October 2008 to February 2014. The research was approved by the Ethics Committee at SCMC, Shanghai Jiao Tong University School of Medicine.

### Genomic DNA library preparation

DNA extraction was carried out using QIAamp DNA Blood Mini Kit (Qiagen, Hilden, Germany, cat. 51106) according to the manufacturer's instruction. Each DNA sample was quantified by agarose gel electrophoresis and Qubit dsDNA BR Assay kit (Life Technologies, Carlsbad, CA, Cat. No. Q32850). Briefly, 1 *μ*g of high‐quality genomic DNA (OD260/280 ratio ranging from 1.8 to 2.0) was diluted with 1× low TE buffer in a 1.5 mL LoBind tube to a total volume of 50 *μ*L and fragmented by sonication on the CovarisS2 (Covaris Company, Woburn, MA). Fragmented DNA was repaired, ligated with Illumina adapters, and size selected, aiming for a 350‐ to 400‐base pair (bp) product. The size‐selected product was then amplified (each sample was tagged with a unique index during this PCR procedure) and validated by using the Agilent 2100 Bioanalyzer (Agilent Technologies, Palo Alto, CA, USA).

### Targeted gene enrichment and sequencing

The amplified DNA was captured with biotinylated oligoprobes (MyGenostics, Baltimore, MD, MyGenosticsGenCap Enrichment Technologies), which were designed to tile along the fusion regions, including exon and intron regions of ETV6 and RUNX1. The capture experiment was conducted according to the manufacturer's protocol. Briefly, the DNA library was mixed with Buffer BL and GenCap gene panel probes (MyGenostics, Baltimore, MD), heated at 95°C for 7 min and 65°C for 2 min on a PCR machine. Twenty‐three microliters of the 65°C prewarmed Buffer HY (MyGenostics) was then added to the mix and held at 65°C with PCR lid heat on for 22 h for hybridization. Fifty microliters of MyOne beads (Life Technologies, Grand Island, NY, USA) were washed in 500 *μ*L 1× binding buffer three times and resuspended in 80 *μ*L of 1× binding buffer. Sixty‐four microliters of 2× binding buffer were added to the hybrid mix and transferred to the tube containing 80 *μ*L of MyOne beads. The mix was rotated for 1 h on a rotator. The beads were then washed with WB1 buffer at room temperature for 15 min once and WB3 buffer at 65°C for 15 min three times. The bound DNA was then eluted with buffer elute. The eluted DNA was finally amplified for 15 cycles using the following program: 98°C for 30 sec (1 cycle); 98°C for 25 sec, 65°C for 30 sec, 72°C for 30 sec (15 cycles); 72°C for 5 min (1 cycle). The PCR product was purified using SPRI beads (Beckman Coulter, Inc, Brea, CA) according to the manufacturer's protocol. The enrichment library was sequenced on Illumina HiSeq2000 sequencer for paired read 100 bp 47, 48.

### Bioinformatics analysis

The sequence variant (SV) detection was completed by using paired‐end sequence data. Insert size distributions were obtained from the mapping results with unimodal insert size distributions. The read pairs should present a mapping quality >30 with a separation distance exceeding four folds of the standard deviation or be in an unexpected orientation. The de novo assembly for all predicted deletions, insertions, inversions, and translocations were performed by using Phrap (http://www.phrap.org/). SAMtools (http://samtools.sourceforge.net/) was used to extract all mapped reads within 500–1000 bp of each predicted breakpoint. Unmapped reads with mates mapping to the SV region were also included. In addition, a read could not have ≥5 bp of unaligned bases on its ends. Reads supported the SV if the read crossed the breakpoint with at least two extra bases and the read did not align to the reference sequence. Positive breakpoints were re‐sequenced by using the Sanger Method.

### Fluorescent in situ hybridization

Air‐dried bone marrow slides from archival samples were used for ETV6‐RUNX1 fusion gene detection using a commercial LSI ETV6/RUNX1 extra signal (ES) Dual Color Translocation Probe (Vysis; Abbott Laboratories Ltd., Chicago, IL), according to the manufacturer's instructions. Briefly, slides were washed in 2× saline/sodium citrate buffer for 2 min at room temperature followed by dehydration in an ethanol series (75%, 85%, and 100%), each for 2 min. Ten microliters of the probe mixture for ETV6‐RUNX1 was spotted on the cell sample. A cover slip was then placed on the probe area and the edges were carefully sealed with rubber solution glue. The 4′,6‐diamidino‐2‐phenylindole (DAPI) antifade (10 *μ*L) was applied on drained slides and developed in the dark for 10 min. Slides were observed with the use of appropriate filters and FISH software. This probe set contains a 350‐kbp probe for the 5′‐end of ETV6 (exons 1–4) and a 500‐kbp probe covering the entire RUNX1 gene.

## Results

### Clinical features of the 26 patients with ETV6‐RUNX1‐positive ALL

Twenty‐six patients with ETV6‐RUNX1‐positive childhood B‐lineage ALL were enrolled in this study, including 20 boys and six girls. The average age was 5.1 years. They were treated with combined chemotherapy, including induction, consolidation, extramedullary prophylaxis, intensification, and maintenance treatment. Four patients presented disease recurrence, two of them relapsed during the maintenance treatment (days 428 and 546, respectively) and the other two relapsed after treatment (days 1297 and 1319, respectively). All cases were registered in the pediatric oncology network database (POND). The clinical characteristics and outcomes are presented in Table 1.

**Table 1:** Clinical characteristics of the 26 patients with ETV6‐RUNX1‐positive childhood ALL

POND number	Gender	Age (years)	WBC (×10^9^/L)	FAB	Blast ratio (%)	Immunophenotype	Fusion gene	Karyotype	Site of relapse	Treatment outcome	Remission time (days)
SCMC‐000206	Male	4.6	158	ALL‐L2	90.4	B	ETV6‐RUNX1	ND	BM; CNS	Relapse	1297
SCMC‐000452	Male	3.2	50.2	ALL‐L3	98.4	B	ETV6‐RUNX1	ND		Alive	1917
SCMC‐000479	Female	6.8	67.6	ALL‐L3	90	B	ETV6‐RUNX1	ND		Alive	1807
SCMC‐000862	Female	1.9	70.2	ALL‐L2	98	B	ETV6‐RUNX1	46,XX		Alive	1594
SCMC‐000863	Male	3.1	7.4	ALL‐L2	97.2	B	ETV6‐RUNX1	46,XY	CNS; BM	Relapse	1319
SCMC‐000864	Male	5.8	5.1	ALL‐L2	75.5	B	ETV6‐RUNX1	46,XY,del(6)(q14q22)^10^/46,XY^2^		Alive	1581
SCMC‐000867	Male	4.3	4.3	ALL‐L1	97.6	B	ETV6‐RUNX1	46,XY		Alive	1576
SCMC‐000926	Male	6.7	119.8	ALL‐L2	95.2	B	ETV6‐RUNX1	ND		Alive	1449
SCMC‐000966	Male	3.9	27.3	ALL‐L2	98.4	B	ETV6‐RUNX1	45,XY,−6,−20,+der(21)t(6;21)(p21;p12)^5^	BM	Relapse	428
SCMC‐001031	Male	13	1.9	ALL‐L3	89.6	B	ETV6‐RUNX1	46,XY		Alive	1301
SCMC‐001108	Female	4.8	3.8	ALL‐L2	97.6	B	ETV6‐RUNX1	46,XX		Alive	1122
SCMC‐001213	Female	2.8	3.6	ALL‐L3	94.8	B	ETV6‐RUNX1	46.XX		Alive	1057
SCMC‐001222	Male	2.4	15	ALL‐L2	97.2	B	ETV6‐RUNX1	46,XY		Alive	1033
SCMC‐001224	Male	6	3.6	ALL‐L2	87.6	B	ETV6‐RUNX1	46,XY		Alive	1034
SCMC‐001245	Male	5.4	15	ALL‐L2	92.8	B	ETV6‐RUNX1	46,XY	BM	Relapse	546
SCMC‐001384	Male	5.9	14.2	ALL‐L3	96.4	B	ETV6‐RUNX1	47,XY,+21^5^		Alive	586
SCMC‐001388	Female	3.9	4	ALL‐L2	92.8	B	ETV6‐RUNX1	ND		Alive	557
SCMC‐001434	Male	3.8	3.6	ALL‐L2	96.8	B	ETV6‐RUNX1	ND		Alive	463
SCMC‐001437	Male	2.3	9.5	ALL‐L2	91.2	B	ETV6‐RUNX1	46,XY		Alive	471
SCMC‐001443	Male	6.1	3.8	ALL‐L3	92	B	ETV6‐RUNX1	46,XY		Alive	466
SCMC‐001478	Female	5.3	3.2	ALL‐L2	93.2	B	ETV6‐RUNX1	ND		Alive	173
SCMC‐001511	Male	4.2	4.2	ALL‐L2	99.2	B	ETV6‐RUNX1	46,XY		Alive	350
SCMC‐001579	Male	7.4	31	ALL‐L3	95.6	B	ETV6‐RUNX1	46,XY		Alive	180
SCMC‐001580	Male	6.7	5.4	ALL‐L2	95.6	B	ETV6‐RUNX1	47,XY,+8^3^/46,XY^17^		Alive	118
SCMC‐001644	Male	9	2.7	ALL‐L3	94.8	B	ETV6‐RUNX1	ND		Alive	174
SCMC‐001645	Male	3	5.3	ALL‐L3	93.2	B	ETV6‐RUNX1	ND		Alive	173

WBC, white blood cell; ND, no data; CNS, central nervous system; BM, bone marrow; POND, pediatric oncology network database.

### Characteristics of ETV6‐RUNX1 breakpoints

**Figure 1:**
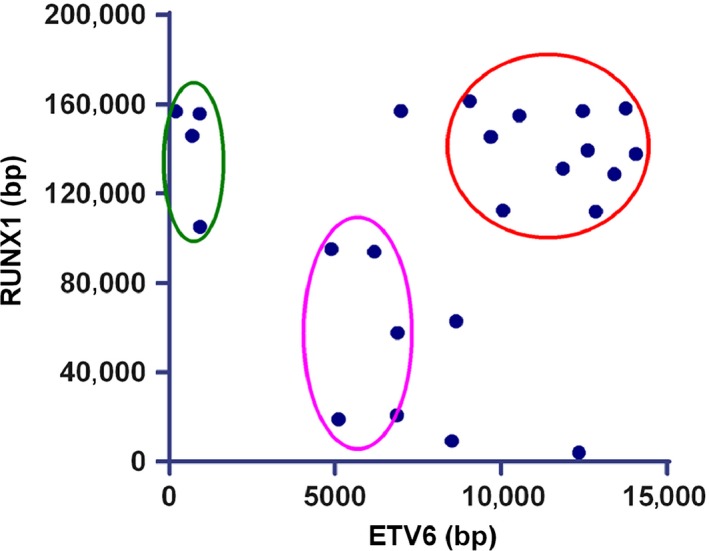
ETV6‐RUNX1 breakpoint clustering regions. The breakpoint clustering regions of all analyzed samples (*n* = 26) are summarized. Breakpoints on both ETV6 and RUNX1 genetic sequence mainly distributed in three areas, which are circled in different colors. The most affected region is circled in red. In addition, some breakpoints gathered in the purple and green circles.

Chr12:12,022,748–12,037,521 and chr21:36,259,140–36,425,395 (*Homo sapiens*, hg19, GRCh37, February 2009) were genomic breakpoint capturing regions of ETV6 and RUNX1, respectively. The target region size was 181 kbp and GenCap probes covered 93.41% of the regions. Target regions were captured and sequenced in all 26 cases of ETV6‐RUNX1‐positive childhood ALL samples. Reads mapping to different chromosomes (i.e., ETV6‐RUNX1, RUNX1‐ETV6, and other related translocations) were confirmed by capillary electrophoresis (Table 2). There were three clustering regions of breakpoints. The most affected region was located from base 8500 to 13,500 and base 100,000 to 160,000 from the beginning of ETV6 intron 5 and RUNX1 intron 1, respectively (Fig. 1).

**Table 2:** Detailed information regarding breakpoints for each patient

Sample	Left_chr	Left_str	Left breakpoint	Gene_left	Right_chr	Right_str	Right breakpoint	Gene_right	Translocation mode
SCMC‐000206	chr12	F	12,023,837	ETV6	chr21	R	36,315,905	RUNX1	c
	chr5	F	5,923,540	KIAA0947 (dist = 433,096)	chr12	F	12,023,848	ETV6	
				FLJ33360 (dist = 387,013)					
	chr21	R	36,315,676	RUNX1	chr5	F	5,684,751	KIAA0947 (dist = 194,403)	
								FLJ33360 (dist = 625,705)	
SCMC‐000452	chr12	F	12,032,593	ETV6	chr21	R	36,275,614	RUNX1	c
	chr12	R	12,032,602	ETV6	chr21	F	36,275,819	RUNX1	d
SCMC‐000479	chr12	R	12,033,450	ETV6	chr21	F	36,266,210	RUNX1	d
	chr12	R	12,029,669	ETV6	chr21	R	36,266,328	RUNX1	b
SCMC‐000862	chr12	F	12,027,788	ETV6	chr21	R	36,326,005	RUNX1	c
	chr12	R	12,026,106	ETV6	chr21	F	36,325,404	RUNX1	d
SCMC‐000863	chr12	F	12,023,597	ETV6	chr21	R	36,275,255	RUNX1	c
	chr5	R	36,122,247	LMBRD2	chr21	F	36,275,263	RUNX1	
	chr5	F	36,122,307	LMBRD2	chr12	F	12,023,436	ETV6	
SCMC‐000864	chr12	F	12,035,255	ETV6	chr21	R	36,416,976	RUNX1	c
SCMC‐000867	chr12	F	12,035,516	ETV6	chr21	R	36,281,679	RUNX1	c
SCMC‐000926	chr12	F	12,023,822	ETV6	chr21	R	362,65,359	RUNX1	c
	chr12	R	12,023,685	ETV6	chr21	F	37,500,446	LOC100133286 (dist = 1503)	d
								CBR3‐AS1 (dist = 3548)	
SCMC‐000966	chr12	F	12,031,429	ETV6	chr21	R	36,411,881	RUNX1	c
	chr6	F	79,662,190	PHIP	chr21	F	36,414,387	RUNX1	
	chr6	R	132,835,666	STX7 (dist = 1322)	chr12	F	12,031,621	ETV6	
				TAAR9 (dist = 23,663)					
SCMC‐001031	chr12	F	12,036,660	ETV6	chr21	R	36,262,972	RUNX1	c
	chr12	R	12,036,598	ETV6	chr21	F	36,263,015	RUNX1	d
SCMC‐001108	chr12	F	120,28,006	ETV6	chr21	R	36,402,216	RUNX1	c
	chr12	R	12,028,066	ETV6	chr21	F	36,402,001	RUNX1	d
SCMC‐001213	chr12	F	120,34,768	ETV6	chr21	R	36,289,889	RUNX1	c
	chr12	R	8,174,744	SLC2A3 (dist = 85,795)	chr21	F	36,289,801	RUNX1	d
				FOXJ2 (dist = 10,558)					
SCMC‐001222	chr12	F	120,35,760	ETV6	chr21	R	36,309,078	RUNX1	c
	chr12	R	12,046,241	ETV6	chr21	F	36,309,775	RUNX1	d
SCMC‐001224	chr12	F	12,029,780	ETV6	chr21	R	36,363,476	RUNX1	c
	chr12	R	12,029,781	ETV6	chr21	F	36,363,464	RUNX1	d
SCMC‐001245	chr12	F	12,036,973	ETV6	chr21	R	36,283,393	RUNX1	c
	chr8	F	26,466,011	DPYSL2	chr12	F	12,037,354	ETV6	
	chr8	R	29,723,210	LOC286135 (dist = 55,819)	chr21	F	36,283,600	RUNX1	
SCMC‐001384	chr12	R	12,036,313	ETV6	chr21	F	36,292,336	RUNX1	d
SCMC‐001388	chr12	F	12,032,965	ETV6	chr21	R	36,308,543	RUNX1	c
	chr12	R	12,033,033	ETV6	chr21	F	36,308,464	RUNX1	d
SCMC‐001434	chr12	F	12,031,547	ETV6	chr21	R	36,358,156	RUNX1	c
SCMC‐001437	chr12	R	12,035,361	ETV6	chr21	F	36,264,128	RUNX1	d
SCMC‐001443	chr12	F	12,029,090	ETV6	chr21	R	36,327,209	RUNX1	c
	chr12	R	12,029,101	ETV6	chr21	F	36,328,064	RUNX1	d
SCMC‐001478	chr12	F	12,029,274	ETV6	chr21	R	36,357,909	RUNX1	c
	chr12	R	12,029,278	ETV6	chr21	F	36,357,982	RUNX1	d
SCMC‐001511	chr12	F	12,029,759	ETV6	chr21	R	36,400,256	RUNX1	c
	chr12	R	12,029,760	ETV6	chr21	F	36,400,256	RUNX1	d
SCMC‐001579	chr12	F	12,029,886	ETV6	chr21	R	38,264,151	RUNX1	c
	chr15	F	86,092,449	AKAP13	chr21	F	36,264,161	RUNX1	
	chr15	R	86,092,248	AKAP13	chr12	F	12,029,986	ETV6	
SCMC‐001580	chr12	F	12,027,748	ETV6	chr21	R	36,261,050	RUNX1	c
SCMC‐001644	chr12	F	12,031,966	ETV6	chr21	R	36,259,665	RUNX1	c
	chr12	R	12,031,976	ETV6	chr21	F	36,259,758	RUNX1	d
SCMC‐001645	chr12	F	12,023,103	ETV6	chr21	R	36,264,307	RUNX1	c

**Figure 2:**
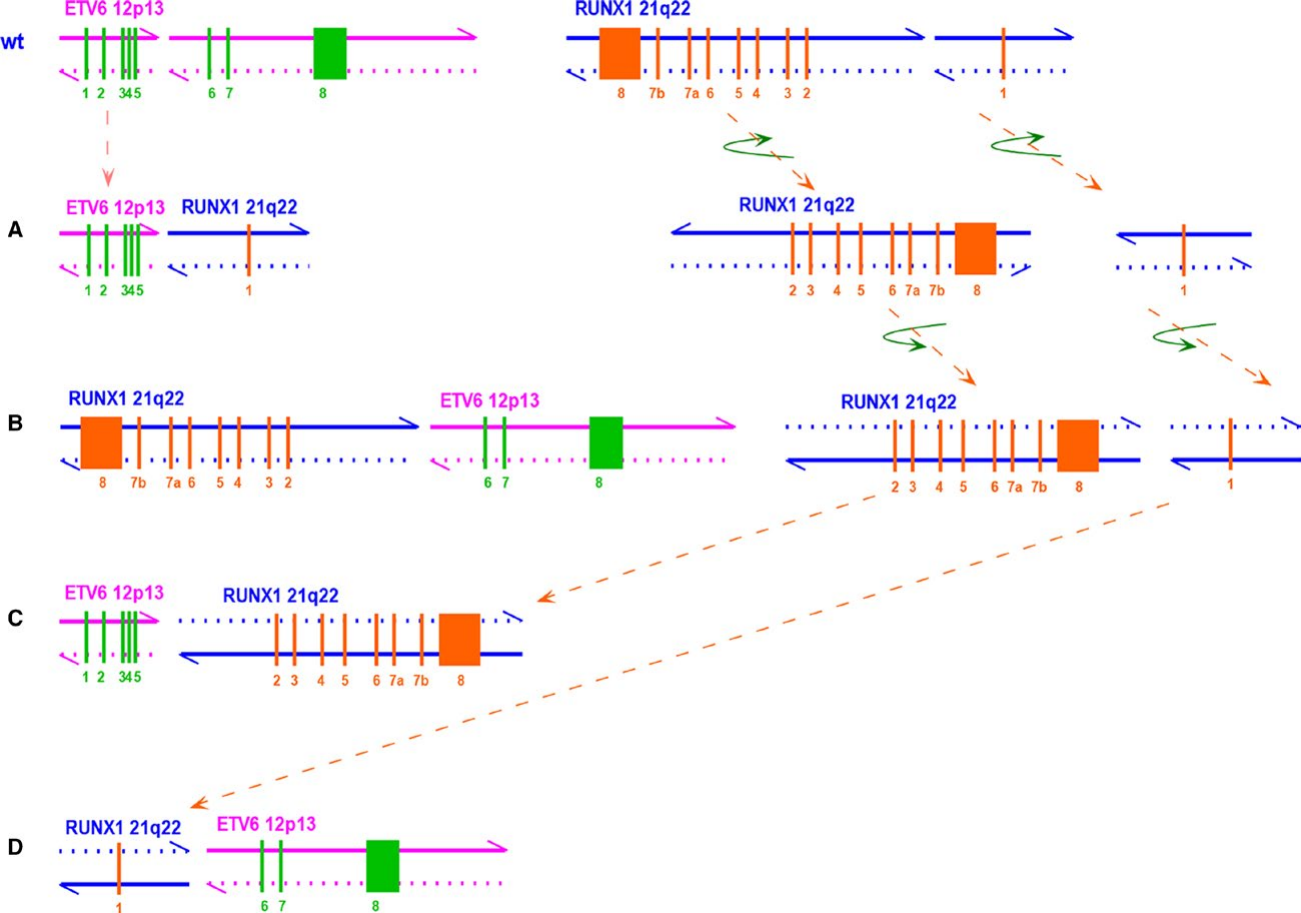
Schematic map of ETV6 wild‐type, RUNX1 wild‐type, and 4 ETV6‐RUNX1 translocation models at the genomic level. Breakpoints are located between exon 5 and exon 6 of ETV6 and exon 1 and exon 2 of RUNX1. Pink and blue arrows show the directions of chromosomes. (A) Intron 5 of ETV6 (including exons 1–5) directly fuses with intron 1 of RUNX1 (exon 1), forward chr12 chain fuses with forward chr21 chain. (B) Intron 1 of RUNX1 (including exons 2–8) directly fuses with intron 5 of ETV6 (including exons 6–8), reverse chr12 chain fuses with reverse chr21 chain. (C) Intron 5 of ETV6 (including exons 1–5) fuses with intron 1 of RUNX1 (including exons 2–8), which has flipped horizontally and vertically, forward chr12 chain fuses with reverse chr21 chain. (D) Intron 1 of RUNX1 (exon 1) flips horizontally and vertically and fuses with intron 5 of ETV6 (including exons 6–8), reverse chr12 chain fuses with forward chr21 chain.

**Figure 3:**
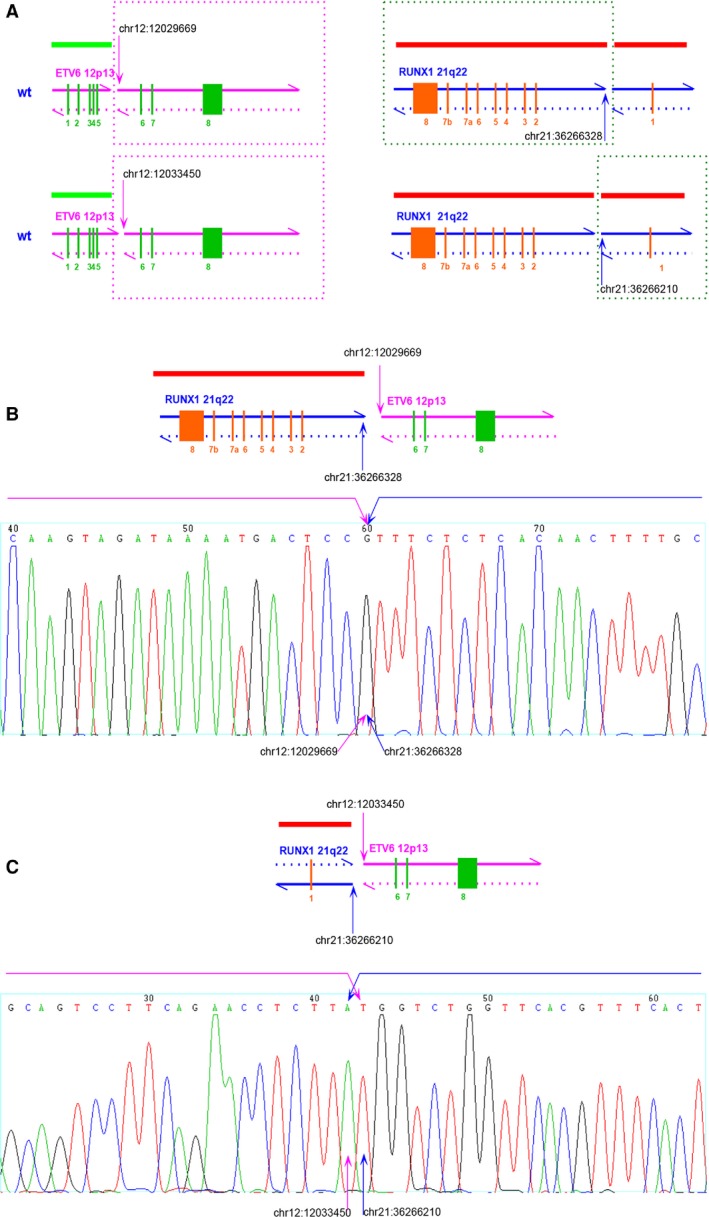
Joint breaks of homologous chromosomes 12 and 21. (A) Two pairs of breakpoint locations were detected in the sample from patient 000479 on ETV6 and RUNX1, respectively. Each chromosome parts in frames participated in the fusion. (B) Patient 00479 presented the type b translocation, which was verified by using the Sanger method. (C) Patient 00479 also presented the type d translocation, which was verified by using the Sanger method.

According to the rearrangement patterns of the genomic flanking sequences for ETV6‐RUNX1, four kinds of translocations should be detected: (1) forward chr12 chain fuses with forward chr21 chain (FF) named type a; (2) reverse chr12 chain fuses with reverse chr21 chain (RR) named type b; (3) forward chr12 chain fuses with reverse chr21 chain (FR) named type c; and (4) reverse chr12 chain fuses with forward chr21 chain (RF) named type d (Fig. 2). The classical ETV6‐RUNX1 translocation (type c), which results in the fusion of ETV6 exons 1 to 5 with almost the whole coding region of RUNX1, was detected in 23 of the 26 patients. Thirteen of these patients simultaneously presented type d. Two patients (patient 001384 and 001437) presented only ETV6‐RUNX1 translocation type d, who ought to have presented type c because the classic ETV6‐RUNX1 had been found at the transcription level by RT‐PCR. In addition, the types b and d coexisted in patient 000479, which suggested that there were joint breaks of homologous chromosomes 12 and 21. Consistent with this, there were two nonidentical breakpoints located in ETV6 and RUNX1 introns simultaneously (Fig. 3).

### ETV6‐RUNX1 three‐way translocation

**Figure 4:**
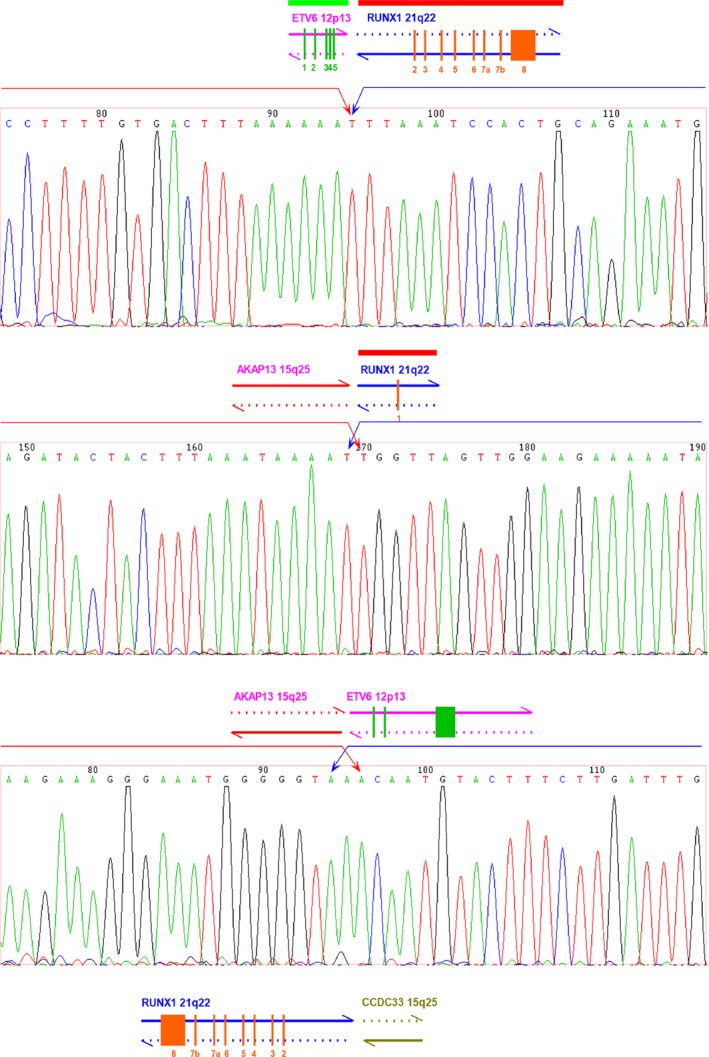
Three‐way translocation of patient 001579. Patient 001579 presented the three‐way translocation‐t(12;15;21)(p13;q25;q22), which involved ETV6‐RUNX1 type c, the translocation of chromosome 15 with ETV6 and RUNX1, respectively. The breakpoint and flanking sequences were sequenced by using the Sanger method.

Beside the two‐way translocation of chromosomes 12 and 21, there were complex three‐way translocations in five of the patients. Among them, two patients (patient 000206 and 000863) presented the translocation of chromosomes 5, 12, and 21 (i.e., t(5;12;21)(p15;p13;q22)), including the classic ETV6‐RUNX1 type c translocation, and the other kind of translocation that the remaining part of ETV6 and RUNX1 fused with chromosome 5. Patient 000966 presented with the t(6;12;21)(q14;p13;q22) translocation, including the classic ETV6‐RUNX1 type c translocation, and the other kind of translocation that the remaining part of ETV6 and RUNX1 fused with chromosome 6. Patient 001245 presented with the t(8;12;21)(p21;p13;q22) three‐way translocation, including the classic ETV6‐RUNX1 type c translocation, and the other kind of translocation that the remaining part of ETV6 and RUNX1 fused with chromosome 8. Patient 001579 presented with the t(12;15;21)(p13;q25;q22) three‐way translocation, including the classic ETV6‐RUNX1 type c translocation, and the other kind of translocation that the remaining part of ETV6 and RUNX1 fused with chromosome 15 (Fig. 4).

More interestingly, four of the five patients with three‐way translocation had already relapsed, while the other 19 patients without three‐way translocations were all in remission. Furthermore, we found that the four patients with three‐way translocation, who relapsed, did relapse after more than 1 year from diagnosis, while the patients with three‐way translocation who did not relapse just received treatment for 6 months. Although the sample size is limited, the Kaplan–Meier analysis also suggested that there may be a significant difference in terms of prognosis between patients with or without three‐way translocations (*P* < 0.0001).

### Clonal origins of ETV6‐RUNX1‐positive ALL relapse

The clonal origins of the leukemia cells from patients with ETV6‐RUNX1‐positive ALL who relapsed were determined by comparing the breakpoint sequences and flanking sequences between paired diagnosis and relapse samples. After detecting the genomic flanking sequences of the breakpoint in the leukemia cells from the patients with ETV6‐RUNX1‐positive ALL who relapsed by the Sanger method, the results indicated that all relapse samples conserved the ETV6‐RUNX1 fusion gene and that the sequences were identical to that of the diagnosis samples. Furthermore, three of the four patients who relapsed conserved the same translocation patterns except for patient 001245, for whom we did not find the translocation of chromosome 8 with ETV6 and RUNX1 (Table 3).

**Table 3:** Comparison of the translocation type between diagnosis and relapse

Patients	Translocation at diagnosis	Translocation at relapse
SCMC‐000206	t(12;21);t(5;12);t(5;21)	t(12;21);t(5;12);t(5;21)
SCMC‐000863	t(12;21);t(5;12);t(5;21)	t(12;21);t(5;12);t(5;21)
SCMC‐000966	t(12;21);t(6;12);t(6;21)	t(12;21);t(6;12);t(6;21)
SCMC‐001245	t(12;21);t(8;12);t(8;21)	t(12;21)

### FISH signal patterns

**Figure 5:**
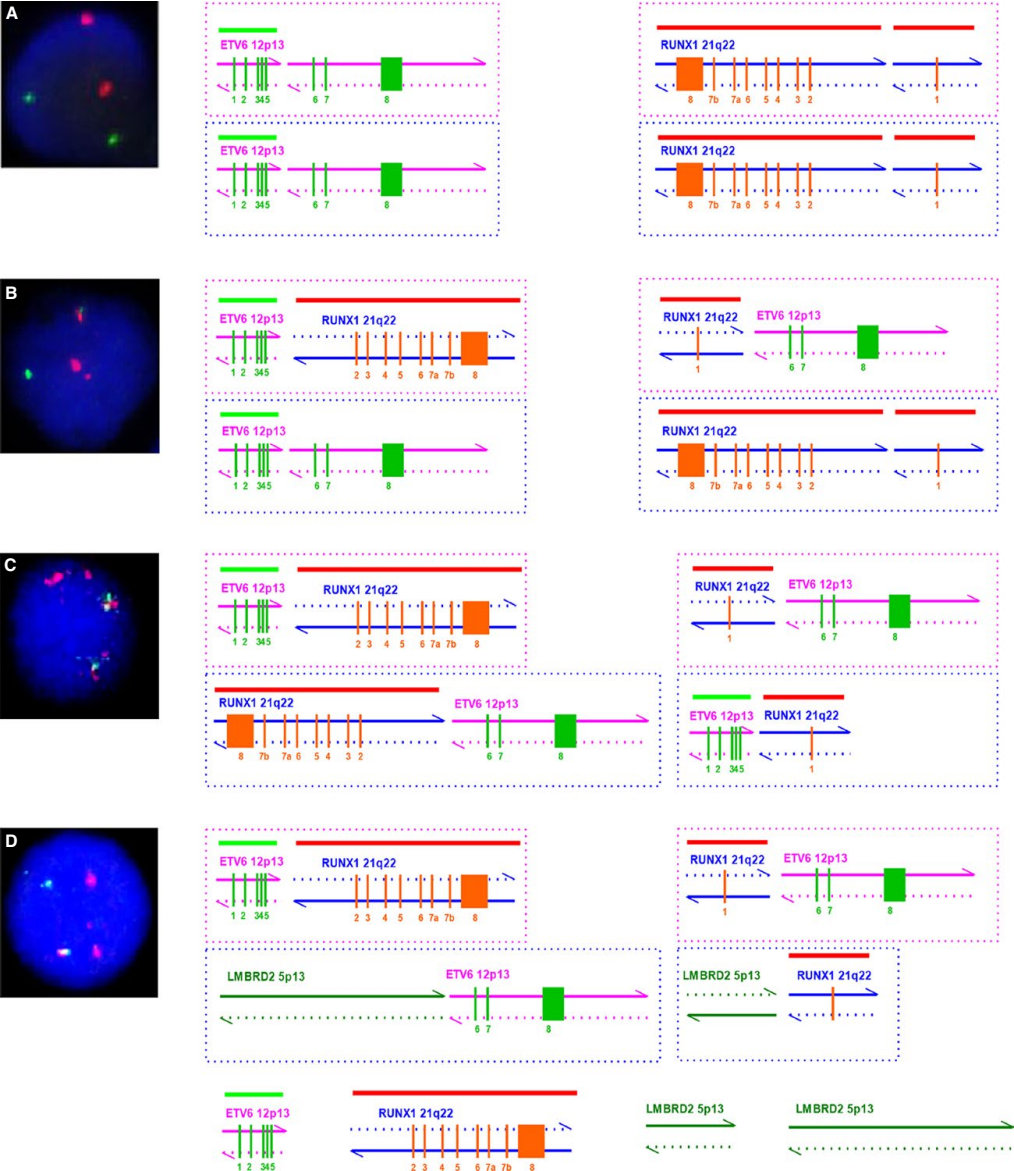
FISH signal patterns. (A) FISH signal pattern for ETV6‐RUNX1‐negative cells is two green and two red. (B) FISH signal pattern for ETV6‐RUNX1‐positive cells is two red, one green, and one yellow. (C) FISH signal pattern for patient 000479 is two red and two yellow. (D) FISH signal pattern for patients with three‐way translocations is three red, one green, and one yellow.

The FISH signal pattern for ETV6‐RUNX1‐negative cells using the Vysis LSI ETV6/RUNX1 ES Dual Color Translocation Probe is two green (ETV6 allele signal) and two red (RUNX allele signal) (Fig. 5A). For ETV6‐RUNX1‐positive cells, the pattern is two red (one large and one small RUNX1 signal), one green (ETV6 allele not involved in the translocation), and one yellow (red and green) fusion signal, corresponding to the ETV6‐RUNX1 fusion gene, which were detected in the classic ETV6‐RUNX1‐positive samples (Fig. 5B). For patient 000479, the pattern was two red (RUNX1 allele signal) and two yellow fusion signals, corresponding to the ETV6‐RUNX1 homozygous fusion genes (Fig. 5C). For patients with three‐way translocations, the pattern was three red (ETV6‐RUNX1 type d, the RUNX1 allele signal was not involved in the translocation, and RUNX1 fused with another chromosome), one green (ETV6 allele was not involved in the translocation), and one yellow fusion signal, corresponding to the type c ETV6‐RUNX1 fusion gene (Fig. 5D).

## Discussion

Genomic DNA from 26 patients with ETV6‐RUNX1‐positive childhood B‐lineage ALL were amplified and captured with biotinylated oligoprobes, and then sequenced on Illumina HiSeq2000 sequencer. According to paired‐end sequence data, mapping results with unimodal insert size distributions, Phrap (http://www.phrap.org/), and SAMtools, patient‐specific ETV6‐RUNX1‐positive breakpoints and flanking sequences were detected in each sample. Positive breakpoints were then verified by using the Sanger method. Using NGS combined with the abovementioned analysis tools, we established a method that allowed us to identify the breakpoints and flanking sequences at the genomic level through the design and synthesis of capturing probes across the genomic regions of the ETV6‐RUNX1 translocation breakpoints. Compared to other methods currently used, this method presents advantages such as the lower amount of genomic DNA used, a relatively lower workload for each sample and the ease of use when analyzing multiple samples simultaneously. Moreover, it provides more meaningful information. Briefly, 1 *μ*g of genomic DNA is sufficient for this method. Furthermore, it is a simple and fast method when compared to inverse PCR or long‐distance PCR, which need tedious molecular biological operations. In addition, many samples can be conveniently handled simultaneously in a single flow cell by using high throughput sequencing and analysis. Furthermore, genomic DNA from other types of samples such as bone marrow smear and dried blood spot can also be used. Finally, detailed breakpoints and flanking sequences can be obtained, which are useful for MRD detecting.

In general, it is very difficult to detect the characteristic of cryptic arrangements by G‐banding analysis 49. Although FISH, by using whole chromosome painting probes or centromeric probes, is a reliable and sensitive molecular method used to identify several chromosomal abnormalities. It cannot provide detailed information regarding ETV6‐RUNX1 breakpoint and cannot discern complicated translocations when there are other translocations involved 50. Similar to FISH, inverse PCR and long‐distance PCR cannot detect other translocations involved besides ETV6‐RUNX1. Inverse PCR is used to amplify DNA with only known sequences and presents two significant disadvantages. First, reasonable sized fragments can only be amplified only an appropriate PCR protocol is designed based on the number of restriction enzymes and the target DNA sequences, which do have restriction sites. Second, most of the genome contains a large number of moderately and highly repetitive sequences, especially in introns. Sometimes, they also exist in unknown function sequences in YAC or Cosmid. Thus, the probes designed for inverse PCR may hybridize with several gene sequences 51. Long‐distance PCR is mainly used for the amplification of large gene fragments. Through multiple sets of primers and analysis of the related results, genetic locations of breakpoints could be determined 52. Based on preliminary information, breakpoints and flanking sequences could be finally determined by long‐distance PCR with newly designed primers and the Sanger method. Using these methods, only known fusion genes can be tested such as ETV6‐RUNX1, while other unknown translocations, involving ETV6 or RUNX1, cannot be detected by only long‐distance PCR. Thus, the method established in this study allows us to avoid the problems mentioned above.

Theoretically, there should be four types of ETV6‐RUNX1 fusion patterns. Among the 26 cases analyzed in this study, one case presented type b, 23 cases were type c, and 16 cases were type d, but no type a was detected. Each chromosome with type c or type d presents one centromere, while chromosomes with type a do not have a centromere and chromosomes with type b have two. Since cells presenting chromosomes without centromeres cannot survive, no type a was detected in our cases. Sixteen cases presented dicentric chromosomes, which could be mitotically stable if one of the two centromeres is inactivated, or if the two centromeres always coordinate their movement to one or the other pole during the anaphase.

Although the prognosis of ETV6‐RUNX1‐positive ALL is favorable following conventional therapeutic strategies, some patients relapse later during or after therapy. Thus, it is crucial to determine the poor prognostic factors at the beginning of the treatment. For these patients, the risk stratification should be adjusted to prevent inadequate treatment. In this study, five patients presented with complicated three‐way translocations including ETV6‐RUNX1. Four of the five patients relapsed at a later stage during the therapy or after the therapy, while the other 21 patients without three‐way translocation are still in remission and the remission time has been more than 1 year for 16 of the 21 patients. Among patients with ETV6‐RUNX1‐positive ALL who experienced late relapse, only one patient with three‐way translocation did not relapse. His remission time is just 6 months, while the other four patients with three‐way translocation relapsed during the second year (day 428) or later. Therefore, further follow‐up is needed. Genomic breakpoints on chromosomes 5, 6, 8, and 15 which we tested were all located in introns, gene LMBRD2 on chr5, PHIP on chr6, DPYSL2 on chr8, and AKAP13 on chr15 were involved. All these genes were broke from the middle into two parts and fused with ETV6 and RUNX1, respectively. LMBRD2 encodes LMBR1 domain‐containing protein 2, which has not been reported any association with leukemia. PHIP protein binds insulin receptor substrate‐1, modulates insulin signaling, and infects pancreatic beta cell growth and survival. DPYSL2 encodes a member of the collapsin response mediator protein family, which form homo‐ and heterotetramers and facilitate neuron guidance, growth, and polarity. This gene may play an important role in Alzheimer's disease. AKAP13 encodes a member of structurally diverse proteins called A‐kinase anchor proteins, which are essential in cell signaling pathways. However, none of these four genes have been reported related to leukemia or cancer. Since the chromosomes which involved in three‐way translocations seemed have no relationship with childhood leukemia, it might be just a phenomenon related to leukemia relapse. According to the above results, the three‐way translocation maybe an important risk factor for relapse in patients with ETV6‐RUNX1‐positive ALL and should be further studied.

For the four patients who relapsed, especially for two who relapsed after therapy (day 1297 and 1319, respectively), whether the relapsed clone is identical to that at diagnosis should be determined. When comparing the pattern of three‐way translocation including ETV6‐RUNX1 breakpoint, the relapsed clone was found to be identical to that detected at diagnosis for three patients, suggesting that the relapsed leukemia clone derived from the dominant clone detected at diagnosis. However, for the other patient (SCMC‐001245), the relapsed clone was not identical to that detected at diagnosis. In fact, the three‐way translocation t(8;12;21) verified by using the Sanger method at diagnosis was not detected in the relapsed sample, only the translocation t(12;21) was verified at relapse. The other translocation of chr8 and chr12/chr21 were not detected at relapse. Although the relapse clone of this patient (SCMC‐001245) was partially different, both the sequences of breakpoints and flanking regions of t(12;21) at relapse were identical to that at diagnosis. Thus, this relapsed clone may be derived from the primary clone detected at diagnosis, while the translocations of t(8;12) and t(8;21) were missed during the proliferation and differentiation of leukemia cells.

Martineau et al. also detected three‐way translocations (chromosomes 12 and 21) by FISH assays, including FISH using whole chromosome paints (wcps) and Multiplex‐FISH (M‐FISH) in combination with the Spectra Vision 24 color chromosome painting kit. In their opinion, variant translocations are prominent among the few relapse karyotypes reported so far 53. We believe that the three‐way translocation should reflect the genetic instability or the extent of genetic damage. There are two distinct levels of gene instability in most tumors. One is at the nucleotide level and is minor. It results in base substitutions, deletions, or insertions of a few nucleotides. The second is at the chromosome level and represent the majority of gene instabilities. It results in losses, gains, and translocations of chromosomes 54. Hence, the three‐way translocation belongs to the latter. Genetic instability is an ongoing process throughout tumor development, which might lead to a genomically heterogeneous population of expanding cells naturally selected for their ability to proliferate, invade distant tissues, and evading host defenses 55. It is reasonable to believe that the higher genetic instability is, the more likely the tumor would relapse. Therefore, the three‐way translocation reflects at least in part the genetic instability and, if the three‐way translocation is an important risk factor for relapse in ETV6‐RUNX1‐positive ALL, it should be further confirmed.

Currently, studies of the recurrent mechanism of ETV6‐RUNX1‐positive B‐lineage ALL concentrate on genetic damages such as CDKN2A/B, CCNC, and other gene deletions. The results show that many deletions are associated with relapse 32. By comparing copy number aberrations (CNAs) on paired diagnostic and relapse samples, researchers found that CNAs were more abundant at relapse than at diagnosis, but only a few cases showed a direct clonal relationship between the diagnosis and the relapse sample 56. However, in this study, relapse samples completely conserved the breakpoints and flanking sequences of the diagnosis samples, which fits the theory that relapses derive from a persistent ETV6‐RUNX1‐positive preleukemic/leukemic clone rather than a resistant leukemia 57. In our opinion, breakpoints and flanking sequences are important markers in clonal evolution of leukemic cells, while CNAs are more likely to be dynamic changes during the whole treatment.

However, the sample size of this study was small, only 26 cases. The sample size needs to be expanded to verify the conclusions of this study and to further our investigation.

## Conflict of Interest

None declared.
